# Diseases of the Rich

**Published:** 1891-11

**Authors:** 


### DISEASES OF THE RICH.

It is an old axiom that health is the greatest of all riches, and it is a thing that money will not buy. Indeed, the possession of great wealth is a predisposing cause of a number of complaints. This is the opinion of Dr. Monin, who has defended it in a book entitled, "Rich Men's Evils." The list of diseases which afflict the rich man is not a long one, but it is, if one may call it so, a substantial one. The possessor of several hundred thousand dollars has the choice between stomach complaints of various sorts, congestions, apoplexies, rheumatisms acute and sub-acute, gout, headaches of every degree, affections of the heart, pleurisy and asthma, liver complaints, jaundice, roseole (from which Queen Victoria suffers), sleepiness, nervous exhaustion, besides a number of distempers which are within the reach of all who can purchase morphine, cocaine and ether.

Whatever your choice may be you may console yourself by the thought that you suffer in good company. Rheumatic people will remember that Mme. de Sévigné, who was a sufferer from it, wore a cheerful countenance and delighted her friends with her sparkling wit. The gouty celebrities form a brilliant host: Horace, Leibnitz, Erasmus, Kant, Franklin, Milton (I give the names as they occur, not in chronological order), Darwin, Sydenham. As you see, doctors are not exempt from it. Literary people are placed by Dr. Monin in company with millionaires, an association no doubt flattering to both, but none the less unlooked for. The weight of their purses, however, has nothing to do with this. But, like plutocrats, they lead a sedentary life, dissipate a quantity of vital energy, and are obliged on that account, to have a substantial diet. Literary men and women are therefore subject to dyspepsia and gout. Gout is a self engendered disease, produced by bad habits of life and food, and can only be cured or checked by the abandonment of such habits. The common habits of life of many people, particularly the well-to-do, engender gout. The quantities of butcher's meat, grease and sugar, salt meat, preserved and canned foods, sweets, and worst of all, beer and alcoholic liquors consumed, directly produce this disease and others of the same nature, but bearing different names. The great thing is to take care of the digestive tract. Every thing that weakens or retards digestion tends to encourage the retention in the body of the poisonous matters which cause diseases of this kind. Take care that the liver is not offended in any way by alcoholic drinks, or it will not perform its work. Still more essential is it to keep up perfect activity of the kidneys, which are important purifiers of the body.

